# Diaphanous related formin 3 knockdown suppresses cell proliferation and metastasis of osteosarcoma cells

**DOI:** 10.1007/s12672-021-00415-8

**Published:** 2021-07-01

**Authors:** Zehua Zhang, Fei Dai, Fei Luo, Wenjie Wu, Shuai Zhang, Rui Zhou, Jianzhong Xu, Qiang Zhou, Lei Song

**Affiliations:** 1 Department of Orthopaedics, First Affiliated Hospital, Army Medical University, No. 30 Gaotanyanzheng street, Chongqing, 400038 China; 2 Department of Orthopaedics, Third Affiliated Hospital, Medical University of Chongqing, Chongqing,, 401120 China

**Keywords:** Osteosarcoma, Diaphanous related formin 3, In vitro, In vivo, Potential therapeutic target, Oncogenic role

## Abstract

Osteosarcoma is a malignant osteoblastic tumor that can gravely endanger the lives and health of children and adolescents. Therefore, there is an urgent need to explore new biomarkers for osteosarcoma and determine new targeted therapies to improve the efficacy of osteosarcoma treatment. Diaphanous related formin 3 (DIAPH3) promotes tumorigenesis in hepatocellular carcinoma and lung adenocarcinoma, suggesting that DIAPH3 may be a target for tumor therapy. To date, there have been no reports on the function of DIAPH3 in osteosarcoma. DIAPH3 protein expression in osteosarcoma tissues and healthy bone tissues adjacent to cancer cells was examined by immunohistochemical staining. DIAPH3 mRNA expression correlates with overall survival and reduced disease-free survival. DIAPH3 protein is upregulated in osteosarcoma tissues, and its expression is significantly associated with tumor size, tumor stage, node metastasis, and distant metastasis. Functional in vitro experiments revealed that DIAPH3 knockdown suppressed cell proliferation and suppressed cell migration and invasion of osteosarcoma cell lines MG-63 and HOS. Functional experiments demonstrated that DIAPH3 knockdown inhibited subcutaneous tumor growth and lung metastasis in vivo. In conclusion, DIAPH3 expression can predict the clinical outcome of osteosarcoma. In addition, DIAPH3 is involved in the proliferation and metastasis of osteosarcoma, and as such, DIAPH3 may be a potential therapeutic target for osteosarcoma.

**Supplementary Information:**

The online version contains supplementary material available at 10.1007/s12672-021-00415-8.

## Introduction

Osteosarcoma, also known as osteogenic sarcoma, is a malignant osteoblastic tumor originating from mesenchymal cells [1, 2]. Osteosarcoma typically occurs in adolescents between 10 and 20 years and substantially endangers the lives and health of those affected. This disease has a complicated genetic background; consequently, its pathogenesis and tumorigenesis are not fully understood. Currently, a combination of multiagent chemotherapy and surgery (e.g., amputation) is the treatment of choice [3]. It is worth mentioning that the prognosis of osteosarcoma has dramatically improved since the development of multiagent chemotherapy. After standard treatment, the 5-year survival rate of patients with osteosarcoma is approximately 65–70% [4]; however, once metastasis occurs, the 5-year survival rate decreases significantly [5]. Therefore, early detection and treatment are essential for reducing osteosarcoma malignancy, and there is an urgent need to identify new and effective biomarkers for osteosarcoma diagnosis. Identification of these biomarkers will facilitate the development of innovative targeted therapies to improve the efficacy of osteosarcoma treatment [6].

Diaphanous related formin 3 (DIAPH3) is a member of the diaphanous subfamily of the formin family, which is involved in microtubule dynamics and actin remodeling, thereby regulating cell movement and adhesion [7, 8]. Hager et al. [9] first suggested that DIAPH3 may be an effective non-canonical regulator of metastasis. DIAPH3 silencing has been shown to increase sensitivity to chemotherapy drugs, such as taxanes and epothilone B [10]. In addition, DIAPH3 expression is higher in hepatocellular carcinoma and lung adenocarcinoma tissues than in normal control tissues [11, 12]. Functional in vitro and in vivo experiments have also suggested that DIAPH3 promotes tumorigenesis in hepatocellular carcinoma and lung adenocarcinoma [11, 12]. These results indicate that DIAPH3 may be a potential target for tumor therapy. Thus, we predicted that DIAPH3 might also be involved in the tumorigenesis of osteosarcoma. To date, no study has investigated the expression and function of DIAPH3 in osteosarcoma.

In this study, we aimed to evaluate the role of DIAPH3 in regulating the progression of osteosarcoma. We sought to highlight the potential role of DIAPH3 as a critical biomarker for diagnosis and prognosis, as well as shed light on the function of DIAPH3 in regulating the proliferation and metastasis of osteosarcoma cell lines in vivo and in vitro.

## Materials and methods

### Patient information and tissue samples

Osteosarcoma tissues (n  =  50) and adjacent normal bone tissues (n  =  50) were obtained from the First Affiliated Hospital of the Army Medical University from 2018 to 2019. All enrolled patients provided detailed clinical information, including age, sex, tumor site, tumor size, and TNM stage. All patients understood the purpose of the study and provided signed informed consent. Our study was approved by the ethics committee of the First Affiliated Hospital of the Army Medical University.

### Bioinformatics analysis

Bioinformatics analysis of DIAPH3 mRNA was performed using gene expression profiling interactive analysis (GEPIA), a web-based tool that provides fast and customizable functionalities based on The Cancer Genome Atlas (TCGA) and Genotype-Tissue Expression (GTEx) data [13].

### Immunohistochemical staining and analysis

DIAPH3 protein expression levels in all osteosarcoma and healthy specimens were examined using immunohistochemical analysis according to the method described in our previous study [14]. Anti-DIAPH3 antibody (ab189373, Abcam, Cambridge, MA, USA) was used at a concentration of 10 µg/mL. Immunohistochemical results were independently measured by two senior pathologists. DIAPH3 staining results were evaluated using the staining intensity score (0, negative; 1, light yellow; 2, brown; 3, tan) and the percentage of positive cells (0,  <  5%; 1, 5–25%; 2, 25–50%; 3, 51–75%; 4,  >  75%). The total score was calculated by multiplying the staining intensity score by the percentage of positive cells. The total scores were grouped into two categories, where a score of 1–4 was regarded as low expression and 5–12 was regarded as high expression.

### Preparation of cell culture

Human osteoblast hFOB 1.19 and human osteosarcoma cell lines (MG-63, HOS, U-2 OS, and Saos-2) were purchased from the Global Bioresource Center at ATCC (Manassas, VA, USA). All the cells were cultured according to the methods specified by the manufacturer’s instructions.

### Construction of DIAPH3 stable knockdown cells

The short hairpin RNA (shRNA) targeting sequence (GCCTGGATATCCAACTTAA) of DIAPH3 (shDIAPH3) was designed and cloned into the pHBLV-U6-MCS-CMV-ZsGreen-PGK-PURO plasmid (Hanbio Biotechnology Co. Ltd, Shanghai, China). Lentiviruses expressing shDIAPH3 (LV-shDIAPH3) and negative control lentiviruses (LV-NC) were packaged by Hanbio Biotechnology Co. Ltd. To construct stable DIAPH3 knockdown cells, MG-63 and HOS cells (1  ×  10^6^ cells) cultured in 6-cm cell culture dishes were infected with LV-shDIAPH3 or LV-NC (multiplicity of infection  =  100). Twelve hours later, culture medium containing lentiviral particles was aspirated, and the cells were screened for infection using a complete culture medium containing puromycin. The culture medium was changed every 2 days for 4 weeks. Cells screened as positive for infection were named as follows: MG-63-NC, MG-63-shDIAPH3, HOS-NC, and HOS-shDIAPH3.

### Quantitative reverse transcription polymerase chain reaction (qRT-PCR)

Total RNA from MG-63-NC, MG-63-shDIAPH3, HOS-NC, and HOS-shDIAPH3 were isolated using TRIzol reagent (Invitrogen, Carlsbad, CA, USA). RNA concentration was quantified using BioPhotometer Plus (Eppendorf, Hamburg, Germany). One microgram of RNA was used for reverse transcription to synthesize cDNA using a M-MLV Reverse Transcriptase Kit (Promega, Madison, WI, USA). A 20 µL PCR reaction system was prepared according to the instructions of the SYBR Green qPCR SuperMix kit (Invitrogen), and the PCR reaction was performed using the ABI PRISM^®^ 7500 Sequence Detection System (Applied Biosystems, Foster City, CA, USA). The internal control was 18S rRNA. The relative expression level of DIAPH3 was calculated using the 2^−ΔΔCT^ method with reference to 18S rRNA. The primers for DIAPH3 and 18S were as follows:

DIAPH3-F: GGGTCACGTTCACACTACAA

DIAPH3-R: CCAAGAAGTTCCGTTTCCTT

18S-F: CCTGGATACCGCAGCTAGGA

18S-R: GCGGCGCAATACGAATGCCCC

### Western blotting

MG-63-NC, MG-63-shDIAPH3, HOS-NC, and HOS-shDIAPH3 cells were harvested, and total protein was isolated using RIPA buffer. After protein quantification, 30 μg of total protein was loaded onto an SDS-PAGE gel. Separated proteins were transferred onto a polyvinylidene fluoride (PVDF) membrane via semi-dry electrotransfer. Membranes were then blocked with 5% (w/v) non-fat dried milk in TBS and incubated with the primary antibody anti-DIAPH3 (ab227276, Abcam, Cambridge, MA, USA) diluted with 5% non-fat dried milk in TBS to appropriate concentrations (1:1000). After washing with TBST three times at 5 min intervals, the membranes were incubated with secondary antibodies. SuperSignal HRP chemiluminescent substrates (Thermo Fisher Scientific, Waltham, MA, USA) were used to produce chemiluminescence, and the signals were exposed to X-rays. All films were scanned, and densitometric analysis was performed using Image Pro-Plus software (version 6.0; Media Cybernetics, Silver Spring, MD, USA). GAPDH was used as the loading control. Relative protein expression was calculated as the relative ratio of the densitometric value of the target protein to the densitometric value of GAPDH. The relative ratio of the target protein to GAPDH in the control group was arbitrarily set to 1.

### Cell counting kit‑8 (CCK8) assays and flow cytometry analysis

For CCK8 assays, MG-63-NC, MG-63-shDIAPH3, HOS-NC, and HOS-shDIAPH3 cells were seeded into 96‐well plates. After seeding for 24, 48, and 96 h, 10 µL CCK8 solution (Beyotime Institute of Biotechnology, Shanghai, China) was added to each well and incubated for 2 h. After incubation, the optical density at 450 nm was measured using a Multiskan MK3 microplate reader (Thermo Fisher Scientific). For flow cytometry analysis, MG-63-NC, MG-63-shDIAPH3, HOS-NC, and HOS-shDIAPH3 cells were seeded into 6‐well plates. After culturing for 48 h, the cells were harvested for apoptosis and cell cycle assays. Apoptosis was detected using the Annexin V-FITC Apoptosis Detection Kit (BD Biosciences, Franklin Lake, NJ, USA). The percentage of apoptotic cells was analyzed using BD FACSDiva version 8.0.1 software. Cell cycle analysis was performed using a Cell Cycle Analysis Kit (Beyotime, Shanghai, China). Cell cycle stages were analyzed using a BD LSRII flow cytometer system (BD LSRII, San Jose, CA, USA).

### Transwell assays

MG-63-NC, MG-63-shDIAPH3, HOS-NC, and HOS-shDIAPH3 cells (1  ×  10^5^ cells) were seeded in the upper chamber of an insert with 200 µL fetal calf serum-free culture medium (pore size, 8 µm) (Becton Dickinson Labware). The lower chamber was filled with 600 µL of the culture medium containing 20% FBS. After incubation for 24 h, cells located in the upper chamber, were removed with a cotton swab, and cells on the underside were fixed with 4% paraformaldehyde at 4 °C for 10 min. Subsequently, the cells were stained with 0.1% crystal violet in 20% ethanol for 20 min. Five randomly selected fields were photographed using a phase-contrast microscope. The cell number in each photograph was counted, and the average migrated cell number per field was calculated. For invasion, the upper chambers were pre-coated with Matrigel (BD Biosciences), and other protocol is same to transwell assay for migration. The assays were performed in triplicate.

### Wound healing assay

MG-63-NC, MG-63-shDIAPH3, HOS-NC, and HOS-shDIAPH3 cells (5  ×  10^5^ cells) were seeded into 6-well plates with straight lines along the bottom of the plate. The following day, a wound was generated across the five straight lines using a sterile 200 μL pipette tip. After washing with PBS to remove fallen cells, the cells were cultured in serum-free medium at 37 °C in a humidified atmosphere with 5% CO_2_. The wound width was monitored and photographed at 24 h. The percentage of wound closure was calculated using the ratio of wound area at 24 h and wound area at 0 h. Wound area was analyzed using Image Pro-Plus software (version 6.0; Media Cybernetics).

### Model construction of subcutaneous xenograft and pulmonary metastasis in nude mice

Sixteen nude mice were divided into four groups (n  =  4) for the construction of subcutaneous xenograft models. Nude mice were subcutaneously injected in the right armpit region with one of the following: MG-63-NC, MG-63-shDIAPH3, HOS-NC, or HOS-shDIAPH3 cells (4  ×  10^6^ cells in 0.2 mL of PBS). At 7, 12, 15, 19, 22, 25, and 28 days after injection, the tumor size was measured using calipers, and tumor volumes were calculated using the formula:

$$ \raise.5ex\hbox{$\scriptstyle 1$}\kern-.1em/ \kern-.15em\lower.25ex\hbox{$\scriptstyle 2$} \, \times \,{\text{L}}\, \times \,{\text{W}}^{{\text{2}}} $$

where L is the length of the tumor and W is the width of the tumor.

To construct a pulmonary metastasis model, MG-63-NC, MG-63-shDIAPH3, HOS-NC, and HOS-shDIAPH3 cells (5  ×  10^5^) were resuspended in 100 μL of Hank’s balanced salt solution and injected intravenously into the tails of nude mice (n  =  4). Six weeks later, all mice were euthanized by intraperitoneal injection of sodium pentobarbital (130 mg/kg). Lung tissues were isolated and fixed in 4% paraformaldehyde. All animal experiments in this study were approved by the ethics committee of the First Affiliated Hospital of Army Medical University.

H&E staining was performed to evaluate the status of lung metastasis using lung tissues isolated from pulmonary metastasis models. To quantitate the number of metastatic nodules in the lung tissues, six randomly selected fields were photographed, and the number of metastatic nodules in each photograph was counted. The average value was defined as the number of metastatic nodules in each group.

### Statistical analysis

Statistical analysis was performed using SPSS software (version 19.0; IBM, Chicago, IL, USA). The results are presented as mean  ±  standard deviation. Statistical comparisons between the two groups were performed using *t *tests. Statistical analysis for more than two groups was performed using one-way analysis of variance (ANOVA) followed by a post-hoc LSD test. The significance of the correlation between DIAPH3 expression and clinicopathological features was determined using the χ^2^ test. Statistical significance was set at *P * <  0.05.

## Results

### DIAPH3 protein level in osteosarcoma tissues and its correlation with clinicopathological features

**Fig. 1:**
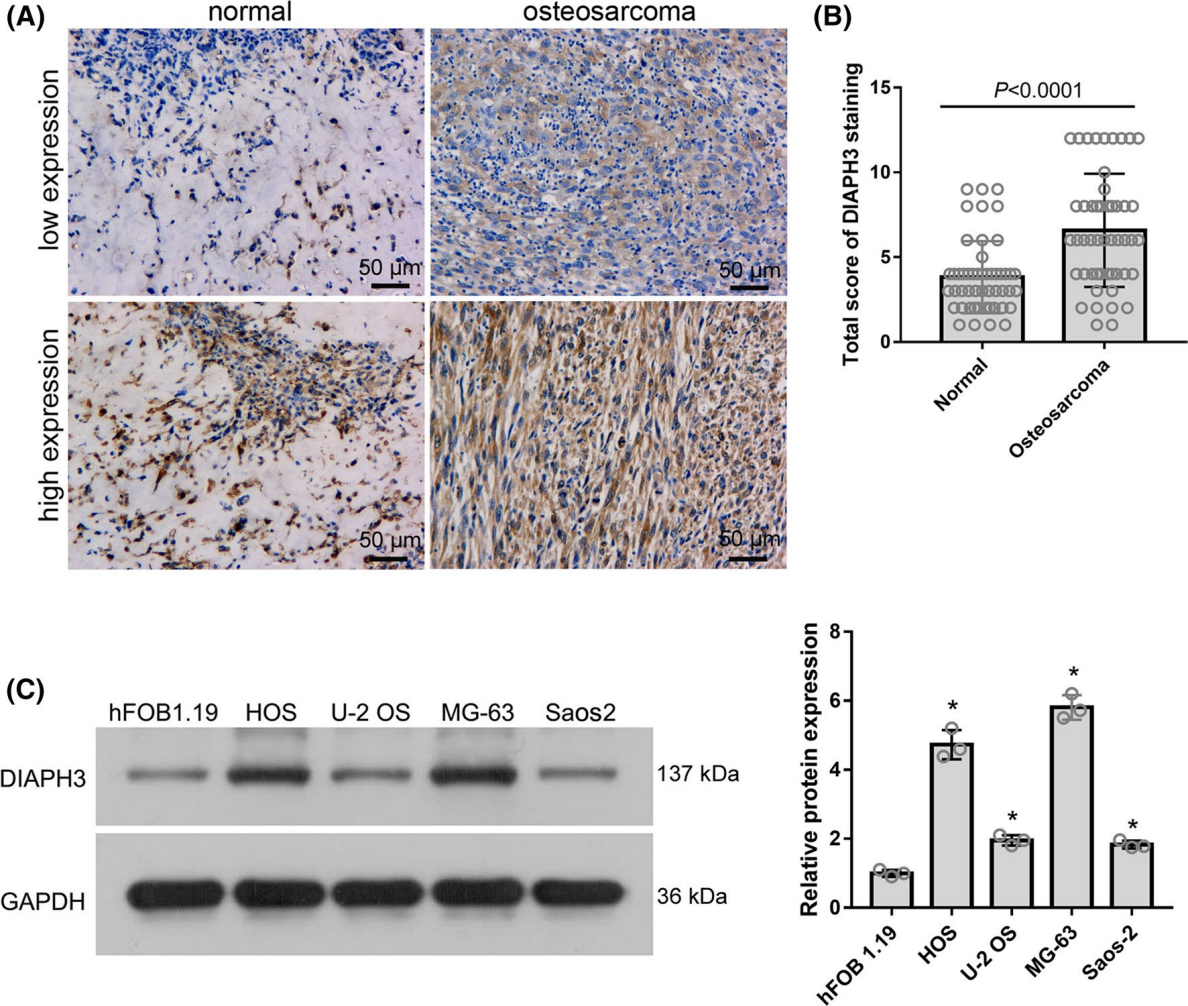
DIAPH3 protein expression was upregulated in osteosarcoma tissues and cell lines. **A** Representative images of low and high DIAPH3 protein expression. **B** Statistical evaluation of the total score of DIAPH3 protein expression based on immunohistochemical staining. **C** DIAPH3 expression in osteosarcoma cell lines and osteoblast hFOB 1.19. On the left are the representative images of western blotting. On the right is the statistical evaluation of relative DIAPH3 protein expression, expressed as a relative ratio of the densitometric value of DIAPH3 protein to the densitometric value of GAPDH. *P  <  0.05, when compared to hFOB 1.19

Immunohistochemistry (IHC) was used to examine DIAPH3 protein expression in 50 osteosarcoma tissues and 50 normal tissues adjacent to the cancer. Representative images of low and high DIAPH3 protein levels are shown in Fig. 1A. IHC results showed high DIAPH3 protein expression in 32 osteosarcoma tissues and ten adjacent normal bone tissues. Statistical evaluation of the total score of DIAPH3 IHC staining showed that DIAPH3 protein expression was significantly higher in osteosarcoma tissues than in normal bone tissues adjacent to the cancer (Fig. 1B). Moreover, DIAPH3 protein expression was higher in the osteosarcoma cell lines MG-63, HOS, U-2 OS, and Saos-2, than in osteoblast hFOB 1.19 (Fig. 1C).

Next, we analyzed the correlation between DIAPH3 protein levels and the clinicopathological features of patients with osteosarcoma. As shown in Table 1, DIAPH3 expression was not associated with age or sex. Furthermore, DIAPH3 expression was significantly associated with tumor size, tumor stage, node metastasis, and distant metastasis.

**Table 1:** Correlation between DIAPH3 protein levels and clinicopathological features of patients with osteosarcoma

Features	n (50)	DIAPH3 protein levels		*P *value
		Low expression (18)	High expression (32)	
Age (years)				
≤ 25	37	12	25	0.375
> 25	13	6	7	
Gender				
Male	29	12	17	0.352
Female	21	6	15	
Tumor size (cm)				
≤ 7.5	24	13	11	0.010
> 7.5	26	5	21	
Tumor stage				
T1 + T2	21	11	10	0.040
T3 + T4	29	7	22	
Node metastasis				
No	28	14	14	0.020
Yes	22	4	18	
Distant metastasis				
No	28	15	13	0.003
Yes	22	3	19	

Bioinformatics analysis using the GEPIA dataset showed that high DIAPH3 mRNA expression was correlated with a poorer overall survival (OS) rate (P  =  0.043) (Fig. S1A) and shorter disease-free survival (DFS) in patients with sarcoma (P  =  0.011; Fig. S1B).

### DIAPH3 knockdown suppressed cell proliferation in vitro

**Fig. 2:**
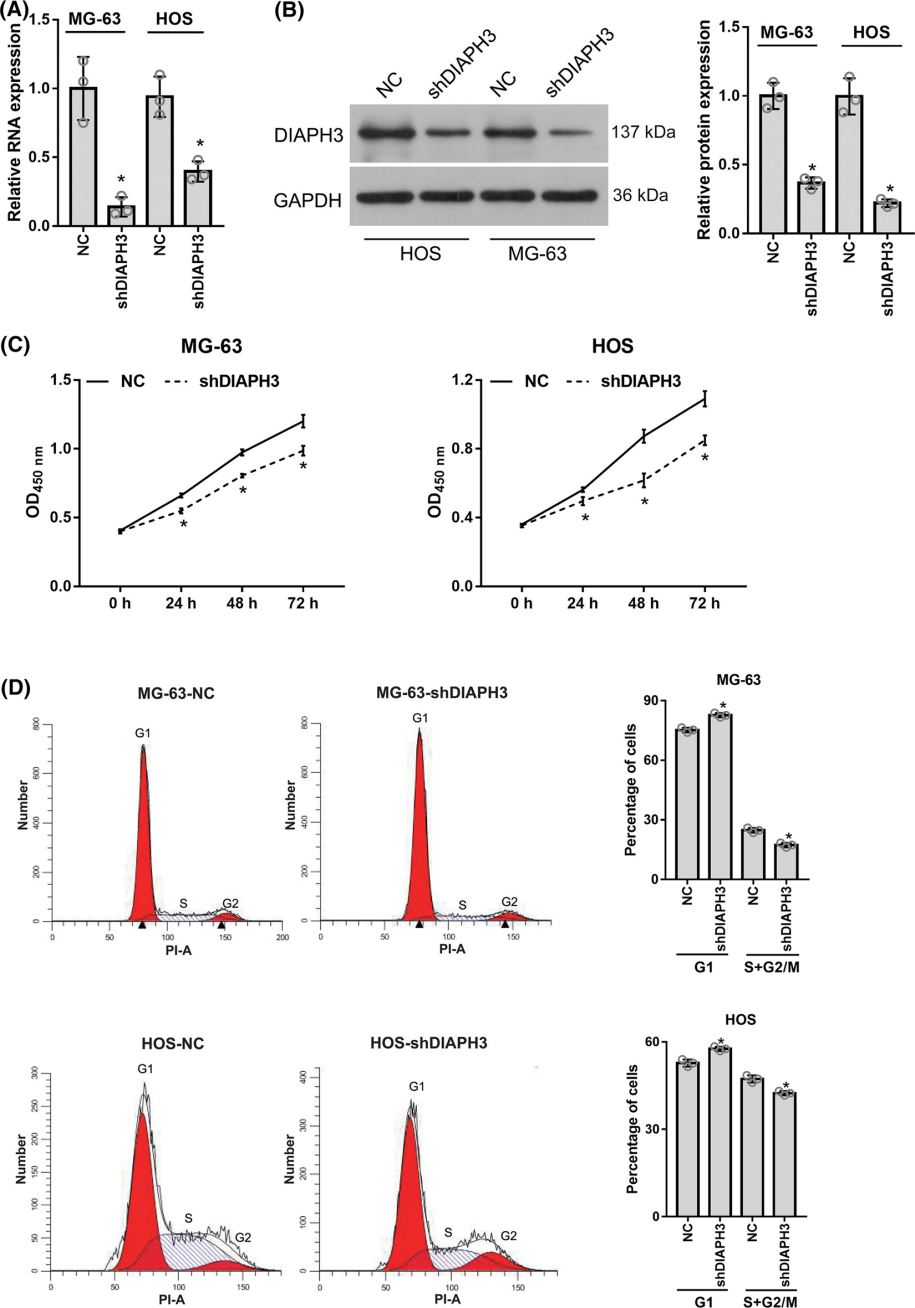
DIAPH3 knockdown suppressed cell proliferation. Stable DIAPH3 knockdown cells, MG-63-shDIAPH3 and HOS-shDIAPH3, and negative control (NC) cells, MG-63-NC and HOS-NC, were harvested. DIAPH3 expression level in MG-63-NC, HOS-NC, MG-63-shDIAPH3, and HOS-shDIAPH3 cells was measured by qRT-PCR (**A**) and western blotting (**B**). **B**, on the left are representative images of western blotting. On the right is the statistical evaluation of relative DIAPH3 protein expression, expressed as a relative ratio of the densitometric value of DIAPH3 protein to the densitometric value of GAPDH. The OD_450 nm_ value of above group cells at 0, 24, 48, and 72 h was ascertained using a CCK8 assay (**C**). The effect of DIAPH3 knockdown on cell cycle was evaluated using flow cytometry analysis (**D**). *P  <  0.05, when compared to NC group

DIAPH3 expression was highest in MG-63 and HOS cells (Fig. 1C). Therefore, stable DIAPH3 knockdown cells were constructed using MG-63 and HOS cells. As shown in Fig. 2A, B, DIAPH3 expression was decreased in MG-63-shDIAPH3 and HOS-shDIAPH3 cells compared to that in MG-63-NC and HOS-NC cells. These results indicate that stable DIAPH3 knockdown cells were successfully constructed.

Moreover, we examined the effect of DIAPH3 knockdown on cell proliferation. The OD_450 nm_ values of MG-63-shDIAPH3 and HOS-shDIAPH3 cells at 48 h and 72 h were significantly lower than those of MG-63-NC and HOS-NC cells (Fig. 2C), indicating that DIAPH3 knockdown suppressed the proliferation of osteosarcoma cells. To investigate the mechanism of DIAPH3 on cell proliferation, the effect of DIAPH3 knockdown on the cell cycle was analyzed. The percentage of MG-63-shDIAPH3 and HOS-shDIAPH3 cells in the G_1_ phase was significantly higher than that in MG-63-NC and HOS-NC cells (Fig. 2D), indicating that DIAPH3 knockdown induced cell cycle arrest in the G_1_ phase of osteosarcoma cells.

The early apoptotic rate was not significantly different between shDIAPH3 and the NC group (MG-63 and HOS) cells (Fig. S2), indicating that DIAPH3 knockdown did not affect the apoptosis of osteosarcoma cells.

### DIAPH3 knockdown suppressed cell migration and invasion in vitro

**Fig. 3:**
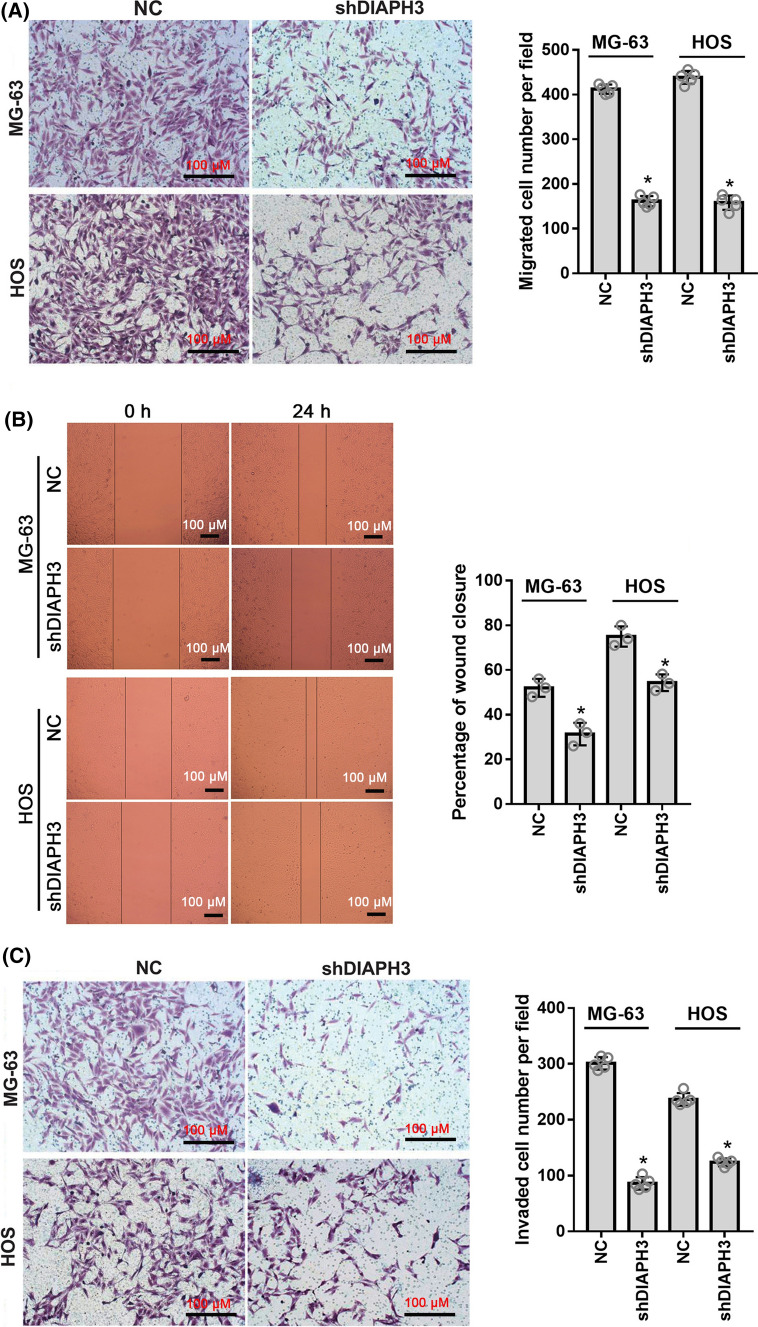
DIAPH3 knockdown suppressed cell migration and invasion. Stable DIAPH3 knockdown cells, MG-63-shDIAPH3 and HOS-shDIAPH3, and negative control (NC) cells, MG-63-NC and HOS-NC, were harvested. The effect of DIAPH3 knockdown on cell migration (**A**, **B**) and invasion (**C**) was evaluated using Transwell or wound healing assays. A representative image is shown on the left. Statistical evaluation of migrating cell number and percentage of wound closure or invaded cell number is shown on the right. *P  <  0.05, when compared to NC group

Transwell and wound healing assays were performed to evaluate the effect of DIAPH3 knockdown on cell migration and invasion. In the Transwell assay, the number of migrated MG-63-shDIAPH3 and HOS-shDIAPH3 cells was significantly lower than that of migrated MG-63-NC and HOS-NC cells (Fig. 3A). In addition, results of the wound healing assay showed that the percentage of wound closure in the MG-63-shDIAPH3 and HOS-shDIAPH3 groups was lower than that in the MG-63-NC and HOS-NC groups (Fig. 3B). These results indicate that DIAPH3 knockdown suppressed the migration of osteosarcoma cells. The number of invaded MG-63-shDIAPH3 and HOS-shDIAPH3 cells in the Transwell Matrigel assay was significantly lower than that of migrated MG-63-NC or HOS-NC cells (Fig. 3C), indicating that DIAPH3 knockdown suppressed the invasion of osteosarcoma cells.

### DIAPH3 knockdown suppressed tumor growth and lung metastasis in vivo

**Fig. 4:**
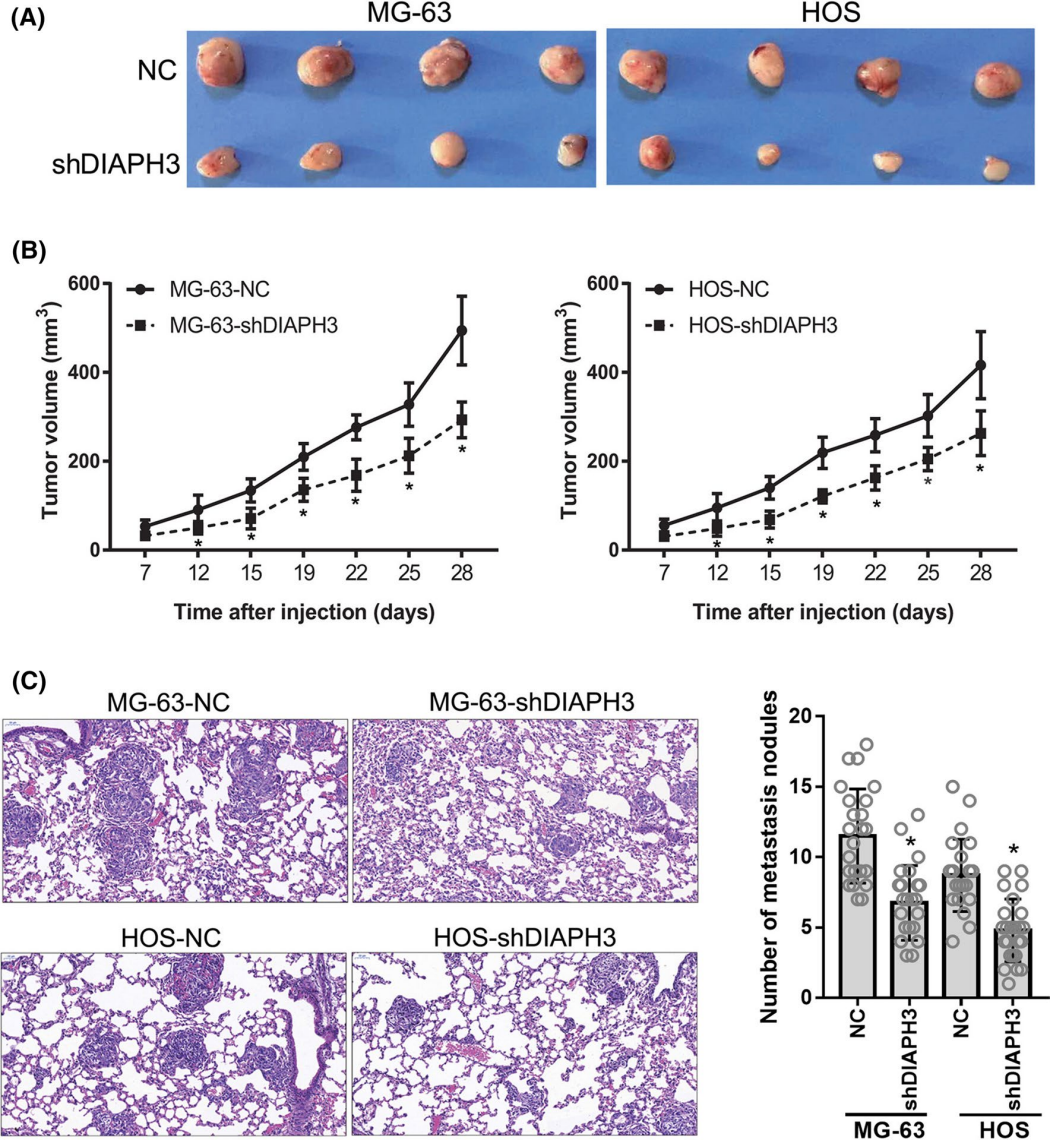
DIAPH3 knockdown suppressed tumor growth and lung metastasis in vivo. Models of subcutaneous xenograft and pulmonary metastasis in nude mice were constructed using stable DIAPH3 knockdown cells, MG-63-shDIAPH3 and HOS-shDIAPH3, and negative control (NC) cells, MG-63-NC and HOS-NC. **A** Photograph of subcutaneous tumors. **B** volume of subcutaneous tumors at 7, 12, 15, 19, 22, 25, and 28 d after cell injection. **C** H&E staining of lung tissues from a pulmonary metastasis nude mouse model. On the left are representative images. On the right is the statistical evaluation of number of metastatic nodules. *P  <  0.05, when compared to NC group

As shown in Fig. S3, DIAPH3 protein expression was decreased in subcutaneous tumors formed by MG-63-shDIAPH3 or HOS-shDIAPH3 cells compared to in that formed by MG-63-NC or HOS-NC cells. These results indicate that DIAPH3 was silenced in vivo. The volume of subcutaneous tumors formed by MG-63-shDIAPH3 or HOS-shDIAPH3 cells was significantly lower than that formed by MG-63-NC or HOS-NC cells (Fig. 4A, B). In addition, the number of metastatic nodules in the lung tissues of nude mice injected with MG-63-shDIAPH3 or HOS-shDIAPH3 cells was lower than that in mice injected with MG-63-NC or HOS-NC cells (Fig. 4C). These results indicate that DIAPH3 knockdown inhibited subcutaneous tumor growth and lung metastasis in vivo.

## Discussion

Previous reports have revealed DIAPH3 as a new therapeutic target for hepatocellular carcinoma and lung adenocarcinoma treatment [11, 12]. Through bioinformatics analysis, we found that high DIAPH3 mRNA expression was correlated with a poorer OS rate and shorter DFS in patients with sarcoma. Sarcoma is a broad family of rare cancers that originate from the soft tissue or bone throughout the body. Therefore, it is rational to hypothesize that DIAPH3 is involved in osteosarcoma progression. In this study, we aimed to verify DIAPH3 expression in osteosarcoma tissues and cells, as well as the effect of DIAPH3 knockdown on cell proliferation and metastasis of osteosarcoma cell lines in vitro and in vivo. These assays will help us to fully understand the function of DIAPH3 in osteosarcoma and identify an effective new target for osteosarcoma.

First, we found that DIAPH3 protein expression was significantly higher in osteosarcoma tissues than in normal bone tissues adjacent to cancer. This disrupted regulation of DIAPH3 suggests that DIAPH3 may play a role in tumorigenesis and the development of osteosarcoma. This hypothesis is further supported by the correlation between DIAPH3 protein levels and the clinicopathological features of patients with osteosarcoma. Upregulation of DIAPH3 expression has also been reported in hepatocellular carcinoma and lung adenocarcinoma [11, 12]. Nevertheless, one study investigating triple-negative breast cancer showed that DIAPH3 expression was significantly decreased in cancer tissues [15]. The inconsistencies of DIAPH3 expression in different cancer types suggest that DIAPH3 may play different roles depending on the type of cancer. Therefore, further investigation of DIAPH3 in other cancers is urgently needed.

Furthermore, functional in vitro and in vivo experiments indicated that DIAPH3 knockdown suppressed the proliferation and metastasis of osteosarcoma cells. We hypothesized that DIAPH3 could be a new target for osteosarcoma, and factors that can suppress DIAPH3 expression or function may be effective for suppressing the tumorigenesis and development of osteosarcoma. Recently, the roles of DIAPH3 in other cancers have also been reported. However, the role of DIAPH3 in different types of cancer varies. DIAPH3 can promote tumorigenesis of hepatocellular carcinoma, pancreatic cancer, and lung adenocarcinoma [11, 16, 17], while suppressing the tumorigenesis of triple-negative breast cancer [15]. The conclusions of our study are consistent with those of most previous reports.

To further understand the mechanism underlying the role of DIAPH3 in cell proliferation, we investigated the effect of DIAPH3 knockdown on the cell cycle. The cell cycle is an essential factor that controls cell proliferation in cancer [18, 19]. We found that DIAPH3 knockdown induced cell cycle arrest in the G_1_ phase. Therefore, we predicted that DIAPH3 is involved in regulating the proliferation of osteosarcoma cells by controlling mitosis. Other mechanisms may also exist. For future studies, a central objective should be to investigate how DIAPH3 affects the proliferation of osteosarcoma cells.

The present study had several limitations. First, the correlation between DIAPH3 protein levels and prognosis was not analyzed because of a lack of follow-up information for the majority of patients enrolled in this study. In future studies, we will incorporate a follow-up plan and enlarge the patient cohort. Second, the regulatory mechanism underlying the involvement of DIAPH3 in the proliferation and metastasis of osteosarcoma has not been elucidated. Recent reports have shown that the downstream mechanisms of DIAPH3 in regulating tumorigenesis are diverse, including interactions with serine/threonine kinase 38 and activation of beta-catenin/T cell factor signaling [11, 17]. It is worth noting that DIAPH3 modulates microtubule stabilization [9]. In amoeboid tumors, DIAPH3 can influence cell mechanics and sensitivity to taxanes by regulating microtubule dynamics [10]. In future studies, we will focus on the possible roles of DIAPH3 in osteosarcoma by regulating microtubule dynamics. In addition, we will focus on the regulatory network of DIAPH3 by measuring mRNA, non-coding RNA, and protein levels.

## Conclusion

DIAPH3 expression predicts the clinical outcomes of osteosarcoma, and DIAPH3 knockdown can suppress cancer cell proliferation and metastasis. Thus, DIAPH3 may be a new potential therapeutic target for osteosarcoma treatment. Moreover, our results suggest that DIAPH3 may play an oncogenic role in osteosarcoma progression. Therefore, in future studies, we will fully elucidate the regulatory mechanisms underlying this process. Our study provides a potential target for pharmaceutical development and novel therapeutic strategies for the control of osteosarcoma proliferation and metastasis involving DIAPH3 inhibition.

## Supplementary Information

Below is the link to the electronic supplementary material.

Supplementary file 1: Figure S1. Results of bioinformatics analysis using GEPIA. GEPIA is a web-based tool used to produce fast and customizable functionalities based on The Cancer Genome Atlas (TCGA) and Genotype-Tissue Expression (GTEx) data. A: Correlation between DIAPH3 mRNA levels and overall survival (OS) rate of patients with sarcoma. B: Correlation between DIAPH3 mRNA levels and disease-free survival (DFS) period of patients with sarcoma. (JPG 256 KB)

Supplementary file 2: Figure S2. DIAPH3 knockdown did not affect apoptosis. Stable DIAPH3 knockdown cells, MG-63-shDIAPH3 and HOS-shDIAPH3, and negative control (NC) cells, MG-63-NC and HOS-NC, were harvested. The effect of DIAPH3 knockdown on apoptosis was evaluated using flow cytometry analysis. On the left, representative images are shown. On the right, statistical evaluation of early apoptotic cell rate (in Q4 quadrant) is shown. * P < 0.05, when compared to NC group. (JPG 484 KB)

Supplementary file 3: Figure S3. DIAPH3 protein level in subcutaneous tumors formed by MG-63-shDIAPH3 or HOS-shDIAPH3 cells. On the left are representative images of western blotting. On the right is the statistical evaluation of relative DIAPH3 protein expression, expressed as a relative ratio of the densitometric value of DIAPH3 protein to the densitometric value of GAPDH. * P < 0.05, when compared to negative control (NC) group. (JPG 118 KB)

## Data Availability

All data from this study are available in this published article.

## Abbreviations

DIAPH3: Diaphanous-related formin 3
GEPIA: Gene expression profiling interactive analysis
GTEx: Genotype-Tissue Expression
shRNA: Short hairpin RNA
LV-shDIAPH3: Lentiviruses expressing shDIAPH3
LV-NC: Negative control lentiviruses
qRT-PCR: Quantitative reverse transcription polymerase chain reaction
CCK8: Cell counting kit‑8
OS: Overall survival
DFS: Disease-free survival

## References

[1] Cortini M, Avnet S, Baldini N (2017). Mesenchymal stroma: role in osteosarcoma progression. Cancer Lett.

[2] Phetfong J, Sanvoranart T, Nartprayut K, Nimsanor N, Seenprachawong K, Prachayasittikul V, Supokawej A (2016). Osteoporosis: the current status of mesenchymal stem cell-based therapy. Cell Mol Biol Lett.

[3] Ritter J, Bielack SS (2010). Osteosarcoma. Ann Oncol.

[4] Siegel RL, Miller KD, Jemal A (2018). Cancer statistics, 2018. CA Cancer J Clin.

[5] Chung LH, Wu PK, Chen CF, Weng HK, Chen TH, Chen WM (2016). Pathological fractures in predicting clinical outcomes for patients with osteosarcoma. BMC Musculoskelet Disord.

[6] Zamborsky R, Kokavec M, Harsanyi S, Danisovic L (2019). Identification of prognostic and predictive osteosarcoma biomarkers. Med Sci.

[7] Katoh M, Katoh M (2004). Identification and characterization of human DIAPH3 gene in silico. Int J Mol Med.

[8] Palander O, Lam A, Collins RF, Moraes TJ, Trimble WS (2021). Non-redundant roles of DIAPHs in primary ciliogenesis. J Biol Chem.

[9] Hager MH, Morley S, Bielenberg DR, Gao S, Morello M, Holcomb IN, Liu W, Mouneimne G, Demichelis F, Kim J, Solomon KR, Adam RM, Isaacs WB, Higgs HN, Vessella RL, Di Vizio D, Freeman MR (2012). DIAPH3 governs the cellular transition to the amoeboid tumour phenotype. EMBO Mol Med.

[10] Morley S, You S, Pollan S, Choi J, Zhou B, Hager MH, Steadman K, Spinelli C, Rajendran K, Gertych A, Kim J, Adam RM, Yang W, Krishnan R, Knudsen BS, Di Vizio D, Freeman MR (2015). Regulation of microtubule dynamics by DIAPH3 influences amoeboid tumor cell mechanics and sensitivity to taxanes. Sci Rep.

[11] Dong L, Li Z, Xue L, Li G, Zhang C, Cai Z, Li H, Guo R (2018). DIAPH3 promoted the growth, migration and metastasis of hepatocellular carcinoma cells by activating beta-catenin/TCF signaling. Mol Cell Biochem.

[12] Xiang G, Weiwei H, Erji G, Haitao M (2019). DIAPH3 promotes the tumorigenesis of lung adenocarcinoma. Exp Cell Res.

[13] Tang Z, Li C, Kang B, Gao G, Li C, Zhang Z (2017). GEPIA: a web server for cancer and normal gene expression profiling and interactive analyses. Nucleic Acids Res.

[14] Dai F, Luo F, Zhou R, Zhou Q, Xu J, Zhang Z, Xiao J, Song L (2020). Calponin 3 is associated with poor prognosis and regulates proliferation and metastasis in osteosarcoma. Aging.

[15] Yi H, Peng R, Zhang LY, Sun Y, Peng HM, Liu HD, Yu LJ, Li AL, Zhang YJ, Jiang WH, Zhang Z (2017). LincRNA-Gm4419 knockdown ameliorates NF-κB/NLRP3 inflammasome-mediated inflammation in diabetic nephropathy. Cell Death Dis.

[16] Rong Y, Gao J, Kuang T, Chen J, Li JA, Huang Y, Xin H, Fang Y, Han X, Sun LQ, Deng YZ, Li Z, Lou W (2021). DIAPH3 promotes pancreatic cancer progression by activating selenoprotein TrxR1-mediated antioxidant effects. J Cell Mol Med.

[17] Xiang G, Weiwei H, Erji G, Haitao M (2019). DIAPH3 promotes the tumorigenesis of lung adenocarcinoma. Exp Cell Res.

[18] Evan GI, Vousden KH (2001). Proliferation, cell cycle and apoptosis in cancer. Nature.

[19] Elmore S (2007). Apoptosis: a review of programmed cell death. Toxicol Pathol.

