# Supplementary figures and images for: Diaphanous related formin 3 knockdown suppresses cell proliferation and metastasis of osteosarcoma cells
Source: Discov Oncol. 2021 Jul 1;12:20. doi: 10.1007/s12672-021-00415-8 (PMC8777534; doi:10.1007/s12672-021-00415-8)

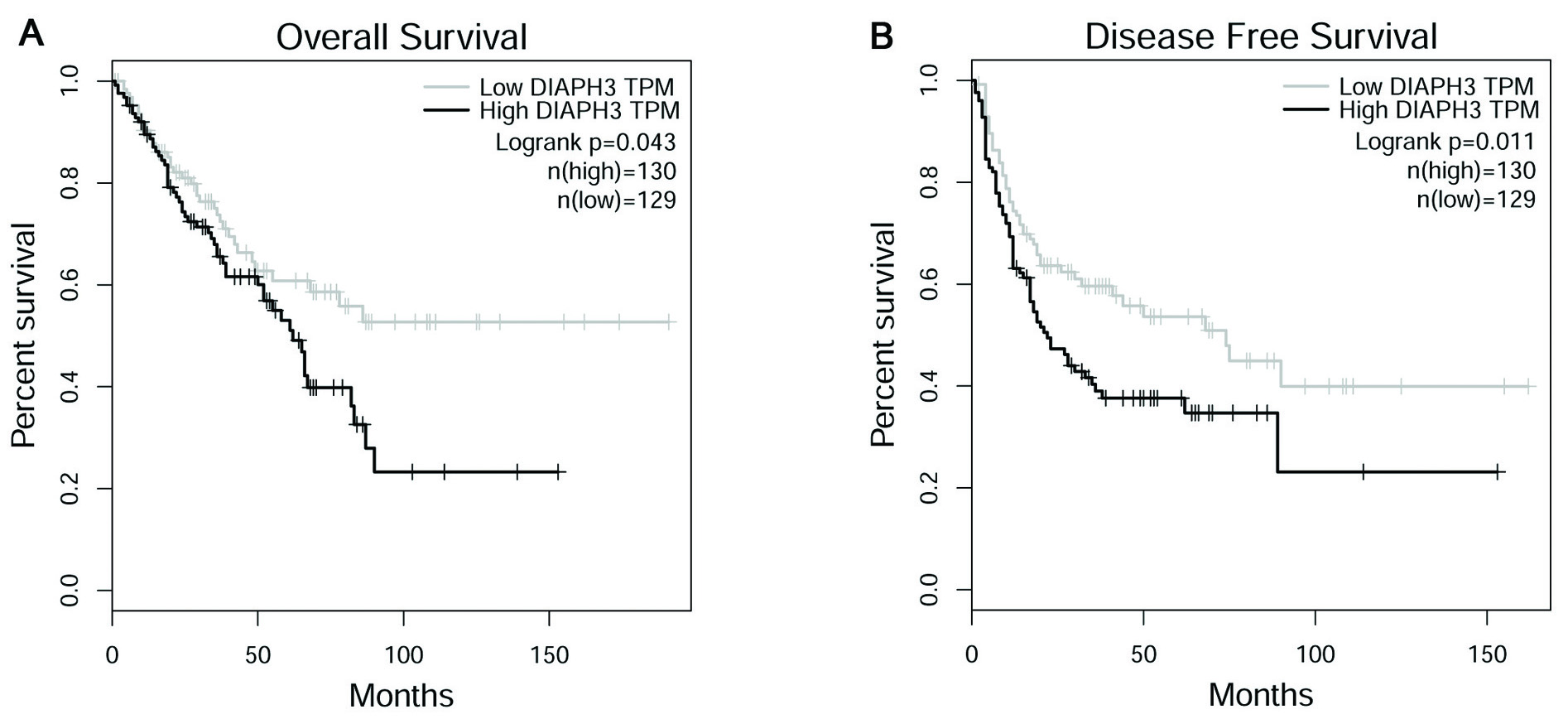

Supplement: Supplementary file 1 — Figure S1. Results of bioinformatics analysis using GEPIA. GEPIA is a web-based tool used to produce fast and customizable functionalities based on The Cancer Genome Atlas (TCGA) and Genotype-Tissue Expression (GTEx) data. A: Correlation between DIAPH3 mRNA levels and overall survival (OS) rate of patients with sarcoma. B: Correlation between DIAPH3 mRNA levels and disease-free survival (DFS) period of patients with sarcoma. (JPG 256 KB) [file 12672_2021_415_MOESM1_ESM.jpg]

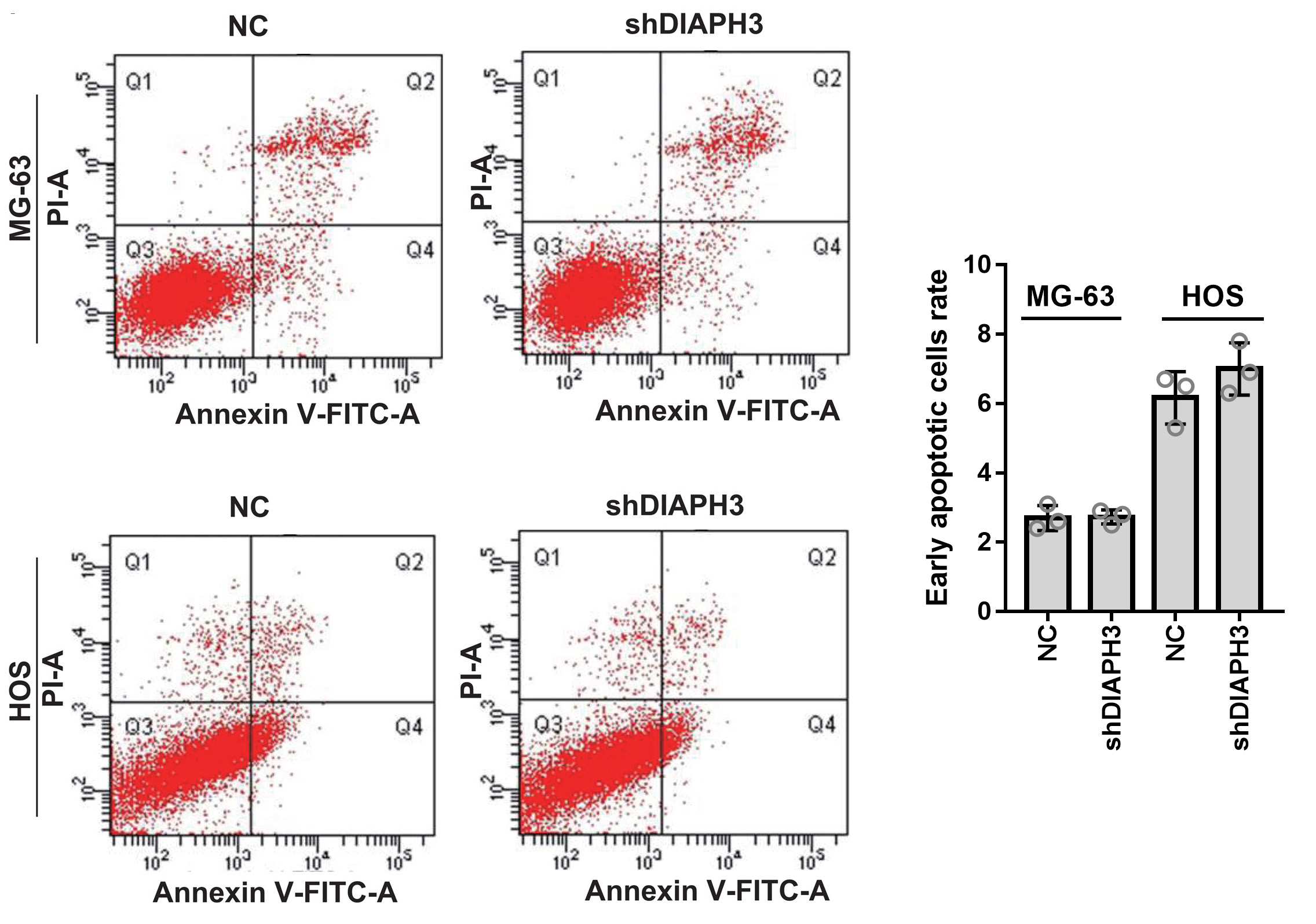

Supplement: Supplementary file 2 — Figure S2. DIAPH3 knockdown did not affect apoptosis. Stable DIAPH3 knockdown cells, MG-63-shDIAPH3 and HOS-shDIAPH3, and negative control (NC) cells, MG-63-NC and HOS-NC, were harvested. The effect of DIAPH3 knockdown on apoptosis was evaluated using flow cytometry analysis. On the left, representative images are shown. On the right, statistical evaluation of early apoptotic cell rate (in Q4 quadrant) is shown. * P < 0.05, when compared to NC group. (JPG 484 KB) [file 12672_2021_415_MOESM2_ESM.jpg]

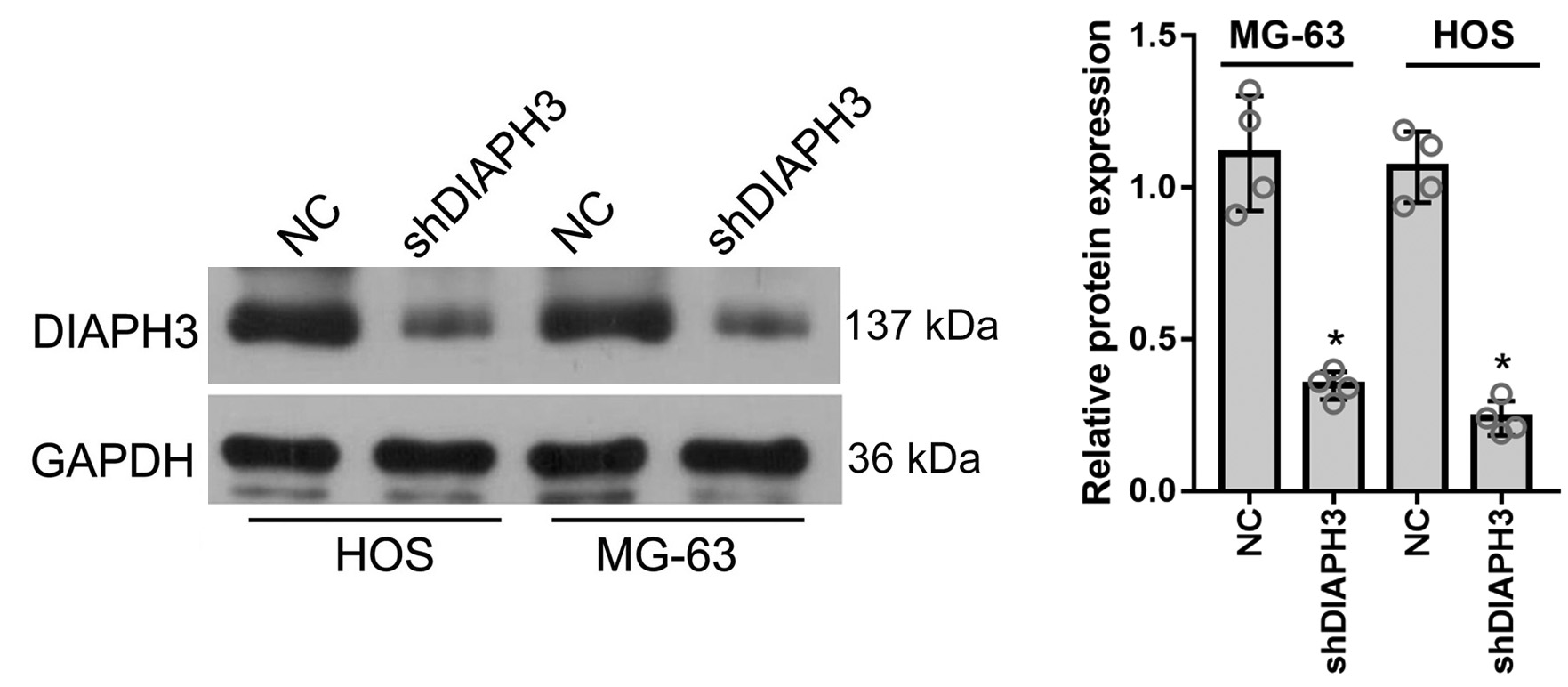

Supplement: Supplementary file 3 — Figure S3. DIAPH3 protein level in subcutaneous tumors formed by MG-63-shDIAPH3 or HOS-shDIAPH3 cells. On the left are representative images of western blotting. On the right is the statistical evaluation of relative DIAPH3 protein expression, expressed as a relative ratio of the densitometric value of DIAPH3 protein to the densitometric value of GAPDH. * P < 0.05, when compared to negative control (NC) group. (JPG 118 KB) [file 12672_2021_415_MOESM3_ESM.jpg]
